# Genetically Modified (GM) Mosquito Use to Reduce Mosquito-Transmitted Disease in the US: A Community Opinion Survey

**DOI:** 10.1371/currents.outbreaks.1c39ec05a743d41ee39391ed0f2ed8d3

**Published:** 2016-05-25

**Authors:** Amesh Adalja, Tara Kirk Sell, Meghan McGinty, Crystal Boddie

**Affiliations:** UPMC Center for Health Security, Baltimore, Maryland, USA; UPMC Center for Health Security, Baltimore, Maryland, USA; UPMC Center for Health Security, Baltimore, Maryland, USA; UPMC Center for Health Security, Baltimore, Maryland, USA

**Keywords:** Aedes, Chikungunya, dengue, GMO, mosquitoes, Zika

## Abstract

Introduction: Mosquito-borne infectious diseases such as dengue, chikungunya, and now Zika, pose a public health threat to the US, particularly Florida, the Gulf Coast states, and Hawaii. Recent autochthonous transmission of dengue and chikungunya in Florida, the recent dengue outbreak in Hawaii, and the potential for future local spread of Zika in the US, has led to the consideration of novel approaches to mosquito management. One such novel approach, the release of sterile genetically modified mosquitoes, has been proposed as a possible intervention, and a trial release of GM mosquitoes is being considered in one Florida community. However, this proposal has been controversial. The objective of this research was to increase understanding of community knowledge, attitudes, and beliefs regarding mosquito control and GM mosquitoes.

Methods: An 18-question self-administered survey was mailed to all households in the identified Key West, Florida neighborhood where a GM mosquito trial has been proposed. This survey was fielded between July 20, 2015 and November 1, 2015. The main outcome variable was opposition to the use of GM mosquitoes. Measures included demographic information and opinions on mosquitoes, mosquito control, and vector-borne diseases.

Results: A majority of survey respondents did not support use of GM mosquitoes as a mosquito control method.

Discussion: Reasons for opposition included general fears about possible harmful impacts of this intervention, specific worries about human and animal health impacts from the GM mosquitoes, and environmental concerns about potential negative effects on the ecosystem. Residents were more likely to oppose GM mosquito use if they had a low perception of the potential risks posed by diseases like dengue and chikungunya, if they were female, and if they were less concerned about the need to control mosquitoes in general. These findings suggest a need for new approaches to risk communication, including educational efforts surrounding mosquito control and reciprocal dialogue between residents and public health officials.

## Introduction

Mosquito-borne infectious diseases have been a continual threat to human health for millennia and, with the recent emergence of Zika virus, have been recognized more fully as a potential concern in the US. Myriad types of mosquitoes spread many different pathogens. Among these mosquitoes the *Aedes* genus serves as the vector for several epidemiologically significant infections including dengue fever, chikungunya, Yellow Fever, and Zika virus.^1^^,^ 2

While mosquito-borne illnesses are often associated with tropical and sub-tropical climates, the temperate climate of the North American continent has in the past supported autochthonous transmission of diseases like malaria and yellow fever.^3^^,^ 4 Prior to Yellow Fever elimination efforts, which targeted the *Aedes aegypti* mosquitoes in the 1960’s, nearly erasing that vector from the Americas, vector-borne Yellow Fever outbreaks occurred seasonally as far north as Boston.^3^ Since the 1980s, vector control efforts have been cut back, or been hampered by a lack of effective pesticides, allowing the* Aedes* species to return to both North and South America and the Caribbean.^5^ Revised vector surveillance estimates by the CDC show that *Aedes aegypti* is present over a wider geographic area in the US than previously thought–as far North as New York and Northern California.^6^ Recently, the *Aedes* vectors have sparked US outbreaks of dengue and chikungunya in which local transmission has occurred, and autochthonous transmission of Zika virus is expected to occur in the US in the coming months.^7^ Such outbreaks have major public health and economic implications that have spurred more aggressive control campaigns in some locations in the Southern US and Hawaii.^8^

In the future, temperature and weather variations have the potential to further expand mosquito habitat ranges and geographic areas with favorable conditions for transmission of vector-borne diseases.^9^^,^ 10 Importantly, concerns about the potential for outbreaks of of Zika virus in the US have grown in recent months, prompting discussion of more aggressive public health and mosquito control responses in the near future.^11^

Vector control activities directly aim to reduce target vector species population numbers. Locales utilize a mix of methods tailored to the specific environment and the particular mosquito’s unique breeding and biting behaviors. These activities can include aerial and ground level insecticide spraying, larvicides, and – crucial to *Aedes* control – elimination of mosquito breeding sites through reduction in standing water.

Effective control of mosquito vectors is difficult to accomplish. Standing water control measures rely on high community compliance to achieve meaningful reductions of breeding sites, which are often located on private property. While this intervention is important for control, it is usually not sufficient. In addition to elimination of standing water, insecticides/larvicides have traditionally been a primary method of mosquito control. However, insecticide use for vector control has, in some cases, sparked concern amongst populations desiring a less “toxic” approach.^12^ The use of some insecticides, like dichlorodiphenyltrichloroethane (commonly known as DDT) – which are effective against mosquitoes, but can be toxic to humans and animals in certain contexts – has diminished.^13^ Additionally, in recent years, *Aedes* mosquitoes in certain regions have become increasingly resistant to some insecticides, rendering this intervention less effective.^14^

One novel approach to *Aedes* control that is increasingly being considered is the release of genetically modified (GM) mosquitoes to diminish populations of wild type *Aedes* mosquitoes.^15^^,^ 16 This technology relies on gene-edited male mosquitoes that are rendered sterile, a characteristic that prevents effective procreation of mosquitoes by outcompeting wild-type males and thereby diminishing population size. Releases of such mosquitoes have occurred in Panama, Brazil, and elsewhere with promising results.^17^^,^ 18

While technologically feasible and seemingly efficacious, GM mosquitoes have generated controversy (as have other GM products) and varied safety-related concerns among the general public.^19^ Lack of opposition, and moreover, community member approval is essential to the success of GM mosquito vector control initiatives.^20^^,^ 21

In order to better understand public perceptions, knowledge and attitudes surrounding vector control and GM mosquito use, we surveyed members of one community in Key West, Florida, where a trial of GM mosquitos is under consideration as a targeted intervention to reduce *Aedes* mosquito populations and protect against vector-borne diseases.^22^ Findings from this study may help public health practitioners and policymakers communicate more effectively with the public around the issue of GM mosquitoes in the future.

## Methods

*Study Design*

Residents in one community in the Florida Keys, in which GM mosquito release is being considered, were surveyed to assess perceptions, knowledge, attitudes and concerns about the use of GM mosquitoes to control vector-borne disease. An 18-question survey, which was developed based on prior surveys and expert opinion, solicited demographic information and opinions on mosquitoes, mosquito control, and vector-borne diseases (Appendix 1). The self-administered survey was mailed to all (456) households in the identified neighborhood and was fielded between July 20, 2015 and November 1, 2015. Of the distributed surveys, 53 were returned as undeliverable. Of the remaining 403 potential household respondents, 22% (n = 89) participated in this study. One response was excluded since very few questions were answered. A total of 88 responses were analyzed.

*Statistical Analysis*

Summary statistics were performed to analyze overall trends in survey responses. The Wilcoxon signed-rank statistical hypothesis test was used to assess whether population mean ranks differed between sub-questions when the survey asked participants to rate different mosquito control methods. Multiple logistic regression was used to model opposition to GM mosquitoes on a number of variables, including demographic information and opinions on mosquitoes, mosquito control, and vector-borne diseases collected for each survey respondent. Simple logistic regression was used to evaluate individual relationships between opposition to GM mosquitoes and collected opinions and demographic information. An expanded set of plausible explanatory variables including all variables that showed significance with simple logistic regression was initially analyzed in a full multiple logistic regression model, but it was determined that a more parsimonious model should be explored due to lack of significance for many variables.

Model selection was done using stepwise selection of variables and analysis of the Akaike Information Criteria (AIC) values. The final model included 3 variables. Because of the high number of unique patterns, the Hosmer-Lemeshow goodness of fit test was the primary regression diagnostic used to show good model fit (p=0.6568). Multi-collinearity was checked using the “collin" command in STATA, which displays several measures of collinearity, including variance inflation factor (VIF). All variables showed low VIF values (from 1.12-2.12) with a mean VIF of 1.59. Data was missing in a small number of responses. For variables included in the regression analysis, the maximum number of missing data points was 6 (6.74%).

*Outcomes*

The main outcome variable was opposition to the use of GM mosquitoes. The survey included the question:

“The Florida Keys Mosquito Control District (FKMCD) is considering using the mosquito control method of introducing male mosquitoes that have been genetically modified (GM) to be sterile in order to reduce the mosquito population in [community]. To what extent do you support of oppose this mosquito control approach in [community]?”

The question included 5 categories of response, including strongly support, support, neutral, oppose, and strongly oppose. These responses were divided into two groups, those who specifically opposed GM mosquitoes (included: oppose and strongly oppose) and those who did not (included all other responses), to create a dichotomous outcome variable. A number of potential independent variables were explored (Appendix 1). After initial data exploration, gender; opinions about mosquito nuisance; worry about mosquito-transmitted diseases; likelihood of contracting those diseases; limitations placed on outdoor activities; and the need to control the mosquito population in the community were used as independent variables in the statistical analysis.

This study was determined to be exempt by the University of Pittsburgh Institutional Review Board (IRB # PRO15060289).

## Results

Between July 20, 2015 and November 1, 2015, 89 households in a Key West neighborhood (22%) responded to our survey. One insufficiently complete response was excluded. Demographic characteristic of 88 respondents included in our analysis are reported in Table 1.

**Table 1: Demographic Data:** 

Question	Responses
Gender (n=87, 99%)	Male (n=40, 46%); Female (n=47, 54%)
Age (n=88, 100%)	18-33 (n=4, 5%); 34-48 (n=13, 15%); 49-64 (n=38, 43%); 65-79 (n=30, 34%); 80 or older (n=3, 3%)
Education (n=85, 97%)	High school degree or equivalent (n=10, 12%); Some college (n=13, 15%); Associate degree (n=14, 16%); Bachelor degree (n=21, 25%); Graduate degree (n=27, 32%)
Residence status	Year round (n=87, 99%); Seasonal (n=1, 1%)
Ownership status (n=86, 98%)	Own home (n=82, 93%); Rent home (n=4, 5%)

*Perceptions about Mosquitoes and Mosquito-borne Diseases*

Respondents were asked whether they perceived mosquitoes to be a nuisance where they live. Forty-nine percent (n=41) of 83 respondents strongly disagreed, disagreed, or were neutral, while 51% (n=42) strongly agreed or agreed that mosquitoes are a nuisance where they live. Sixty-nine percent of residents (n=60), reported that they rarely or never limit their time outdoors due to mosquitos while 9% (n=27) responded that they sometimes or often limit their time outdoors because of mosquitoes. This question was followed with a yes/no question about whether there is a need to reduce the mosquito populations in their community. Most residents who responded answered “yes” (n=67, 79%)

Participants were also asked about their level of worry about mosquito-spread diseases like dengue fever or chikungunya. Of the 87 respondents to this question, 32 (37%) reported that they “haven’t thought about it” or were “not worried at all,” while a majority (n=55, 63%) were either “a little worried” or “very worried.” This question was followed by a question asking whether residents had known anyone who had been sick with dengue fever or chikungunya. Of the 87 responses to this question, 65 (75%) answered “no” they hadn’t known anyone with these diseases, and 22 (25%) answered “yes.” Residents were then questioned about perceived likelihood that they or someone they know would contract dengue fever or chikungunya while living in their community. A large majority (n=75, 85%) of the 88 total respondents to this question thought that it was “very unlikely,” “unlikely,” or “uncertain” that anyone they knew would contract these diseases. Only 13 (15%) respondents answered that it was “likely” or “very likely” to happen.

*Perceptions about Mosquito Control Methods*

The survey next asked a succession of questions about mosquito control methods. First, residents were asked to rate a series of mosquito control methods that might be applicable to their community. Respondents were asked to rate each question from 1-5 (1 being the method they supported the most, and 5 being the method supported the least). Based on the mean score of each control method, residents were most supportive of the method of “draining standing water on private property to reduce mosquito breeding” (mean=1.98), followed by treating standing water with larvicides (mean=2.49), spraying pesticides at ground level (mean=3.01), spraying insecticides in the air (mean=3.14), and residents were least supportive of using “GM sterile male mosquitoes to reduce the mosquito population (mean=4.14). The difference in distributions of opinions regarding eliminating standing water from private property and the use of GM mosquitoes to control the mosquito population was found to be statistically significant using the Wilcoxon signed rank test (z=-5.57, p=<0.0001).

*Perceptions about GM Mosquitoes*

Residents were then asked directly to what extent they support using GM mosquitoes for control in their community. Of the 86 who responded, 50 (58%) said they either “oppose” or “strongly oppose” GM mosquito use, while 36 (42%) were either neutral or “support” or “strongly support” GM mosquito use for vector control in their community. The question about GM mosquito use was followed up with two questions aimed at understanding the reasons behind why they either supported or did not support this control option. Residents were asked to check all reasons that applied. For those who supported or were neutral on GM mosquito use, the survey provided 4 possible reasons for support of this method. Of these reasons for supporting GM mosquito use, respondents chose one reason most often: “GM mosquitoes could reduce the need to use pesticides/larvicides in [community] for mosquito control” (n=24). Other reasons, including reduction in pesticide-resistant mosquitoes (n=16) concern about mosquito-transmitted disease (n=15) and reduction of mosquito nuisance (n=14), were chosen less often (See Figure 1).

**Frequency of Chosen Reasons for Supporting GM Mosquito Use:**
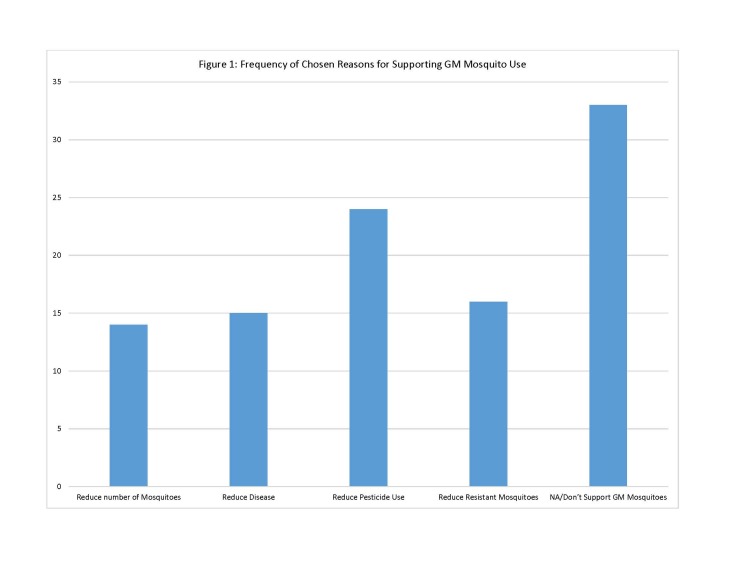

For those residents who did not support GM mosquito use, the survey provided 7 possible reasons for opposition of this method. Of these reasons, respondents chose the following reasons most often: “I am concerned about the overall safety of GM mosquitoes” (n=37); “Introduction of GM mosquitoes could upset the local ecosystem by eliminating mosquitoes from the food chain” (n=20); and “The use of GM mosquitoes could lead to the use of other GMO products in [community]” (n=19). Other reasons included concerns that the mosquito could make people sick and/or pass on modified genes to people and animals, but were chosen less and thus were less important to the respondents (See Figure 2).

**Frequency of Chosen Reasons for Opposing GM Mosquito Use:**
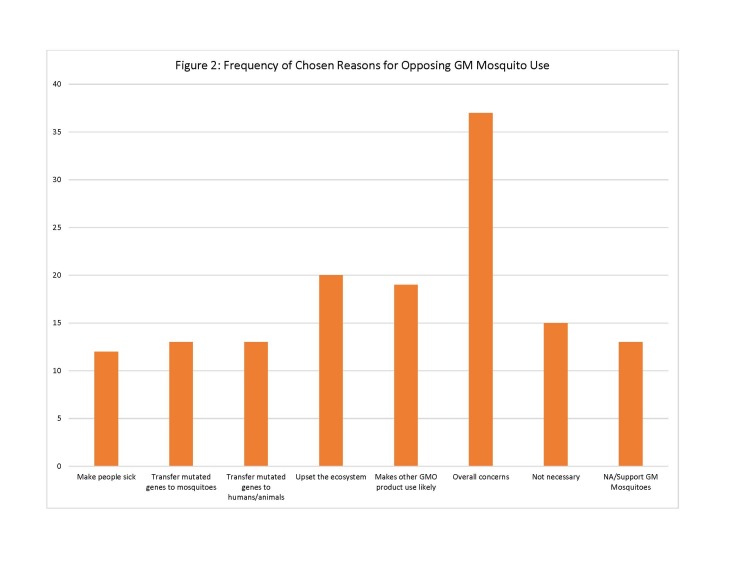

Residents were asked to characterize how much they had read, heard or seen about the use of GM mosquitoes as a method of mosquito control prior to receiving this survey. A large majority (n=60, 83%) had been exposed to this information “some” or “a lot,” while only 12 (17%) of the residents who responded had read, heard, or seen “not much” or “nothing” on this topic. Finally, the survey asked residents about their opinion of the Florida Keys Mosquito Control District (FKMCD), which is responsible for mosquito control in the community of interest. Overall, most (n=74, 87%) respondents’ had either “very favorable,” “favorable” or “neutral” opinions toward the District, while a minority (n=11, 13%) had “unfavorable” or “very unfavorable” opinions of FKMCD.

*Characteristics associated with opposition to GM mosquitoes*

Beyond summary statistics, we sought to assess relationships between opposition of GM mosquito use in vector control (dependent variable) and other demographic factors and opinions. First, simple regression was used to evaluate individual relationships between opposition to GM mosquito use and demographic variables. In this analysis only gender was found to be significantly associated with GM mosquito opposition at the p<0.05 level (OR=5.0, p=0.001), with females more likely to oppose use than males.

We then sought to evaluate the relationship between GM opposition and other questions about mosquito control and risk perception. Using simple logistic regression, we found significant associations between GM mosquito opposition and opinions about mosquito nuisance, worry about mosquito-transmitted diseases, likelihood of contracting those diseases, limitations placed on outdoor activities, and the need to control the mosquito population in the community (Table 2). Each of these variables were negatively associated with GM mosquito opposition. If residents considered mosquitoes to be a nuisance, if they worried about dengue and Chikungunya or thought that contracting those diseases was likely, limited outdoor time because of mosquitoes or thought that mosquito control was very important, they were less likely to be opposed to GM mosquitoes. When multiple logistic regression was applied to the same set of variables, only gender (OR=7.47, p=0.002) and Disease likelihood (OR=7.47, p=0.01) had significant associations with GM mosquito opposition at the p<0.05 level (Table 2).

**Table 2: Crude and Adjusted Relative Odds of GM Mosquito Opposition.*Adjusted model includes Gender, mosquitoes as nuisance, disease worry, disease likelihood, limit outdoor time, and need for control:** 

	Crude			Adjusted*		
Variable	OR	95% CI	P value	OR	95% CI	P value
Gender (female)	5.00	(1.97-12.67)	0.001	7.47	(2.14-26.16)	0.002
Perception of mosquitoes as a nuisance	0.47	(0.29-0.75)	0.002	0.81	(0.42-1.56)	0.520
Worry about mosquito-transmitted diseases	0.37	(0.19-0.74)	0.004	0.82	(0.31-2.18)	0.697
Perception of likelihood of acquiring disease	0.38	(0.22-0.65)	<0.001	0.38	(0.18-0.80)	0.010
Limit outdoor time because of mosquitoes	0.59	(0.36-0.96)	0.033	0.90	(0.43-1.90)	0.780
Need for mosquito control	0.19	(0.05-0.73)	0.016	0.38	(0.07-1.96)	0.249

Because of the lack of significance for many of the variables in the initial adjusted model, a subsequent model was developed. The final model included 3 variables: opinion about the likelihood of contracting a mosquito–transmitted disease, gender, and opinion about the need to control the mosquito population in the community. In this final model, opinion about likelihood of contracting a mosquito–transmitted diseases (OR=0.31, p=0.001) and gender (OR=6.93, p=0.001) were both significantly associated with opposition to GM mosquitoes , with those who considered the likelihood of acquiring a mosquito-borne disease to be low and females being more likely to oppose GM mosquitoes. Opinion about the need to control the mosquito population in the community as a variable was found to be borderline significantly associated with GM mosquito opposition (OR=0.23, p=0.055), with those who supported a greater need to control the mosquito population more likely to support GM mosquitoes (Table 3).

**Table 3: Crude and Adjusted Relative Odds of GM Mosquito Opposition: Final Model. *Adjusted final model includes Gender, disease likelihood, and need for control.** 

	Crude			Adjusted Final*		
Variable	OR	95% CI	P value	OR	95% CI	P value
Gender (female)	5.00	(1.97-12.67)	0.001	6.93	(2.13-22.58)	0.001
Perception of likelihood of acquiring disease	0.38	(0.22-0.65)	<0.001	0.31	(0.16-0.60)	0.001
Need for mosquito control	0.19	(0.05-0.73)	0.016	0.23	(0.05-1.03)	0.055

## Discussion

Our work represents a baseline evaluation of the level of support or opposition to GM mosquitoes in an area in which their use is being contemplated because of recent experience with both dengue and chikungunya autochthonous transmission. Importantly, this evaluation took place during a time period before Zika virus became a concern and provides foundational information to judge potential changes in attitudes over time and with the introduction of new concerns about mosquito borne diseases. Not surprisingly, the general cultural animus against most things labeled as “genetically modified” extends to genetically modified mosquitoes. However, the nuances of that opposition merit further study and incorporation into public outreach, discussion, dialogue and education efforts surrounding this issue.

Of interest is the marked gender-split with females more likely to be opposed to the use of GM mosquitoes than males. One hypothesis is that this stance reflects broader concerns about genetically modified products that have been shown to be heightened among women, and potential concerns about possible effects on children and future generations.^23^^,^ 24 ^,^ 25 ^,^ 26 ^,^ 27 However, we did not query subjects about the presence of children in their household. A similar gender-skew has been found in studies of vaccine acceptance, with females being less accepting than males.^28^

The finding that a person’s estimation of the likelihood of contracting dengue or chikungunya strongly influences their acceptance or rejection of GM mosquitoes as a control option is also of particular interest. This finding has strong face-validity in that risk perception strongly influences the countermeasures people will support in a variety of situations.^29^ Most of our respondents thought it unlikely that they or someone they know would contract one of the listed illnesses, and only a minority of respondents believed local disease transmission to be risk. This finding is puzzling because dengue and chikungunya cases have occurred in Key West in recent years. Local outbreaks have been covered extensively by the local media and the local community was blanketed with information aimed at community engagement, as our group noted in a prior study.^8^ It is unclear whether the knowledge of the prior outbreaks was extinguished in the minds of many respondents or whether risk perception has just waned since these cases occurred.

An important extension of this work would be evaluate the impact of the 2015-2016 Zika virus outbreak in the Western Hemisphere on awareness, risk perception, and attitudes. Zika is a virus also transmitted by *Aedes* type mosquitoes. The Zika virus outbreak, occurring in a post-West African Ebola context, has received a very high level of media attention. In Florida, the Governor recently declared public health emergencies in 4 counties due to Zika concerns, despite there being no evidence of autochthonous transmission in the state to date.^30^ In contrast, no emergency declarations occurred in 2009, or in subsequent years, when there has been autochthonous transmission of dengue in several Florida counties,^31^ or in 2014, when the first locally acquired chikungunya case was identified in Florida.^32^ Zika virus, with its association with adverse fetal and neonatal outcomes, may change the risk perception of those that live in *Aedes*-laden regions. Adverse pregnancy outcomes might also be a factor in shifting females’ perceptions as they recalibrate risks of *Aedes*-associated infections with new information about Zika and its potential threats to children. In a recent nationwide survey by Purdue University, 78% of participants supported the use of GM mosquitoes to fight Zika virus.^33^ While the Purdue study surveyed a much wider population, it may provide an indication of potential changes in public support for GM mosquito use that may emerge as communities face the threat of Zika virus transmission in their neighborhoods. In contrast however, a different nation-wide survey published in February by the University of Pennsylvania’s Annenberg Public Policy Center found that 35% of respondents believed that GM mosquitoes cause the spread of Zika,^34^ a belief which may reduce local support for GM mosquitoes.

*Limitations*

The primary limitations of this study are a potential for self-selection bias and non-response bias in the group of respondents. It may be that individuals who were in greater opposition to or more in support of GM mosquito use were more likely to respond to this survey. Thus the survey may not be representative of the beliefs and attitudes of the community at large. This potential limitation may make this study more representative of those residents with strong positive or negative feelings about GM mosquito use. While this is a limitation, the survey still provides insight into how and why motivated and vocal members of this Florida community might support or oppose these vector control options, and can be useful to inform future public health approaches to gathering support for GM mosquitoes or other alternative methods of mosquito control.

## Conclusion

We present the first community-based survey of resident attitudes towards GMO mosquitoes in a region of a U.S. state in which *Aedes*-associated infectious disease risks are extant. Gender and individual risk-perception were found to be highly predictive of support or opposition to GM mosquito use. These findings may help public health practitioners and policymakers to improve communication and dialogue with the public around potential use of GM mosquitoes in the future, which may be increasingly important as new mosquito-borne threats such as Zika emerge.

## Competing Interest Statement

The authors have declared that no competing interests exist.

## Data Availability Statement

A link to the survey data can be found here: http://www.upmchealthsecurity.org/our-work/pubs_archive/pubs-pdfs/2016/GM%20Mosquito%20Data.pdf

## Appendix: Key Haven Mosquito Control Opinion Survey

1) Your Participant ID is: ___________

2) What is your Gender (choose 1 of the following choices)?

• Male

• Female

• Other

3) Do you live in Key Haven permanently, seasonally, or are you just visiting (choose 1)?

• Year round permanent resident (this is my primary residence year round)

• Seasonal resident (I live here less than year round or this is not my primary residence)

• Visitor (I don’t live here, I’m just visiting)

4) If you live in Key Haven year round or seasonally, do you own or rent the property in which you reside on Key Haven (choose 1)?

• I own a home in Key Haven

• Renter

• Other (Please specify)

5) What is your age (choose 1)?

• Less than 18 Years Old

• 18-33 Years Old

• 34-48 Years Old

• 49-64 Years Old

• 65-79 Years Old

• 80 Years Old or Older

6) What is the highest level of school you have completed or the highest degree you have received (choose 1)?

• Less than high school degree

• High school degree or equivalent (e.g., GED)

• Some college but no degree

• Associate degree

• Bachelor degree

• Graduate degree

7) To what extent do you agree with the statement: “Mosquitoes are a nuisance where I live” (choose 1)

• Strongly agree

• Agree

• Neutral

• Disagree

• Strongly disagree

8) How worried are you about dengue fever, chikungunya, and other mosquito-spread diseases (choose 1)?

• Very worried

• A little worried

• Not worried at all

• Haven’t thought about it

9) Do you know anyone who has gotten sick with dengue fever or chikungunya (choose 1)?

• Yes

• No

10) How likely do you think it is that you or someone you know will get dengue fever or chikungunya while you live in Key Haven (choose 1)?

• Very likely

• Somewhat likely

• Uncertain

• Unlikely

• Very unlikely

11) How often do you limit your time outdoors because of mosquitoes (choose 1)?

• Often

• Sometimes

• Rarely

• Never

12) Do you think there is a need to control or reduce mosquito populations in Key Haven (choose 1)?

• Yes

• No

13) If you think mosquito control is needed, rank the mosquito control methods you would support in Key Haven from 1-5 (1 being the method you would support the most, 5 being the method you would support the least).

• Spraying pesticides from the air to kill adult mosquitoes

• Spraying pesticides at ground level to kill adult mosquitoes

• Introducing genetically modified (GM) sterile male mosquitoes to reduce the mosquito population

• Draining standing water on private property to reduce mosquito breeding

• Treating standing water with larvicides to kill young mosquitoes

14) What is your opinion of the Florida Keys Mosquito Control District (FKMCD)?

• Very Favorable

• Favorable

• Neutral

• Unfavorable

• Very Unfavorable

15) The Florida Keys Mosquito Control District (FKMCD) is considering using the mosquito control method of introducing male mosquitoes that have been genetically modified (GM) to be sterile in order to reduce the mosquito population in Key Haven. To what extent do you support or oppose this mosquito control approach in Key Haven?

• Strongly support

• Support

• Neutral

• Oppose

• Strongly oppose

16) If you support or do not oppose the use of genetically modified (GM) mosquitoes in Key Haven, please select the reason(s) for your support from the list below (check all that apply).

• GM mosquitoes could reduce the number of mosquitoes and thus reduce the nuisance of mosquitoes in Key Haven

• GM mosquitoes could reduce the chance that I or someone I know or love will get a mosquito-transmitted disease like dengue fever or chikungunya

• GM mosquitoes could reduce the need to use pesticides/larvicides in Key Haven for mosquito control

• GM mosquitoes could reduce the emergence of pesticide-resistant mosquitoes

• Not applicable, I do NOT support the use of GM mosquitoes

17) If you oppose or do not support the use of genetically modified (GM) mosquitoes in Key Haven, please select the reason(s) for your opposition from the list below (check all that apply).

• GM mosquitoes could bite me or someone I know or love and cause them to become sick

• GM mosquitoes might pass on modified genes to the wild mosquito population

• GM mosquitoes might pass on modified genes to people or animals

• Introduction of GM mosquitoes could upset the local ecosystem by eliminating mosquitoes from the food chain

• The use of GM mosquitoes could lead to the use of other GMO products in Key Haven

• I am concerned about overall safety of GM mosquitoes

• Use of GM mosquitoes is unnecessary because dengue and chikungunya are not serious threats in Key Haven

• Not applicable, I support the use of GM mosquitoes

18) Before today, how much had you read, heard, or seen about the use of genetically modified (GM) mosquitoes as a method of mosquito control?

• A lot

• Some

• Not Much

• Nothing
